# Short-Term Prognostic Predictive Evaluation in Female Patients With Ischemic Stroke: A Retrospective Cross-Sectional Study

**DOI:** 10.3389/fneur.2022.812647

**Published:** 2022-03-24

**Authors:** Fettah Eren, Cihat Ozguncu, Serefnur Ozturk

**Affiliations:** School of Medicine, Selcuk University, Konya, Turkey

**Keywords:** stroke, gender, prognosis, female, women, short term

## Abstract

**Introduction and Aim:**

Stroke is a disease with high mortality and morbidity. Although studies are generally performed on all patients with stroke, it is known that gender has an effect on etiology and prognosis. This study aimed to determine the importance of clinical stroke scales and laboratory markers in determining the short-term prognosis of female patients with ischemic stroke of anterior circulation.

**Materials and Methods:**

The study was planned as a retrospective and cross-sectional study. SEDAN score, the National Institutes of Health stroke scale (NIHSS), the Modified Rankin Scale (mRS), the Glasgow Coma Scale (GCS), and THRIVE score applied to the patients at the time of admission were recorded. Admission blood glucose, hemoglobin, leukocyte, urea, albumin, and blood lipid levels were evaluated. The relationship of all these parameters with in-hospital prognosis, mortality, and disability at discharge was examined. The relationship between groups and data was analyzed using the SPSS package program after the normality analysis.

**Results:**

In this study, there were 733 female patients with stroke with a mean age of 69.53 ± 14.51 years and 858 male patients with stroke with a mean age of 64.27 ± 13.29 years. Hospitalization time, length of stay in the intensive care unit, ventilation need rate, mortality, and dependency rate were higher in female patients (*p* = 0.001). The NIHSS, SEDAN, and THRIVE scores were higher in female patients who had in-hospital mortality, had a poor prognosis, and who were discharged as dependent (*p* = 0.001). GCS was lower in this patient group (*p* = 0.001). Blood glucose, creatinine, leukocytes, urea, and CRP levels were higher; the albumin and hemoglobin levels was lower in female patients who had fatal outcomes (*p* = 0.009, 0.001, 0.001, 0.001, 0.001, and 0.020; respectively). In female patients who were dependent at discharge, blood urea, glucose, and CRP levels were higher and the albumin levels were lower than those in female patients who achieved functional independence (*p* = 0.001, 0.016, 0.002, and 0.001, respectively).

**Conclusion:**

Our study showed that the short-term prognosis is worse in female patients who had an ischemic stroke of anterior circulation. It also revealed some clinical and laboratory parameters that could predict this situation. More intensive monitoring may be needed to improve prognosis in female patients.

## Introduction

Stroke is a cerebrovascular disease that causes temporary or permanent effects and causes various neurological findings (1). Every year, varying degrees of disability and mortality occur in millions of people worldwide due to stroke. In a recent study, it was revealed that ~80.1 million people had a stroke, and more than half of these patients were women (2). Considering the personal, social, and familial effects of stroke, it is important to identify and eliminate the risk factors. Gender is one of the most important risk factors for stroke. The effect of gender varies with age. The incidence of stroke is higher in childhood and early adulthood for men (3). Stroke rates increase in women in middle age due to hormonal factors (4). The incidence of stroke in advanced age exceeds that of men (3). Conditions such as age, comorbid diseases, and, lifestyle complicate the assessment of the effect of gender on stroke.

Many studies show that stroke-related mortality is higher in female patients, as well as disability; however, there are also studies stating that gender does not have an independent effect on mortality (5–7). Furthermore, it should be considered that women have a stroke at an older age when mortality rates are also high; therefore, it is difficult to establish a relationship between mortality and gender, to predict prognosis, and to identify markers with prognostic predictive value (8). There are studies showing the prognostic predictive value of systemic inflammatory cells, blood glucose, and blood lipid parameters in stroke (9, 10). However, these markers are easily affected by many systemic conditions, especially those related to hormonal factors. The aim of this study was to determine the in-hospital prognostic predictive value of disability scales and laboratory and clinical parameters in female patients with stroke.

## Materials and Methods

This study was planned as a retrospective and cross-sectional study. Selcuk University Clinical Researches Local Ethics Committee was obtained before the study (Ethics committee approval: 2020-473). Female patients who received inpatient treatment with a diagnosis of stroke in the Neurology Clinic of Selcuk University Medical Faculty Hospital between 2016 and 2020 were included in the study; 2,030 patients with stroke over the age of 18 were screened. Patients with a diagnosis of head trauma, intracranial hemorrhage, intraventricular or subarachnoid hemorrhage, subdural or epidural hematoma, and sinus vein thrombosis were excluded from the study. Patients with ischemic stroke of posterior circulation were also excluded from the study. Data from 611 female patients with acute ischemic stroke in the anterior circulation were included for the study. The short-term prognosis of 683 male patients followed during this period was also evaluated following the same criteria.

The patients' age, accompanying chronic diseases (diabetes mellitus, hypertension, coronary artery diseases, malignancy, chronic renal failure, dementia, hyperlipidemia, cardiac arrhythmia), and smoking were evaluated. Consciousness levels, muscle strength, presence of cranial nerve involvement, dysarthria and aphasia were evaluated. SEDAN score, the National Institutes of Health stroke scale (NIHSS), the Modified Rankin Scale (mRS), the Glasgow Coma Scale (GCS), and THRIVE score applied to the patients' record at the time of admission were recorded. The SEDAN score includes five parameters, including age, basal blood glucose, finding of early infarction, and hyperdense artery sign on CT. The GCS is especially used for assessing the state of consciousness; the mRS, the NIHSS, and THRIVE scores evaluate the current disability (11–15). Patients were divided into groups according to the following treatments: intravenous thrombolysis; endovascular thrombectomy; intravenous thrombolysis + endovascular thrombectomy.

The blood results of the patients at the time of hospitalization were analyzed. Reference blood glucose (mg/Dl), leukocytes (K/Ul), urea (K/ul), albumin (g/Dl) and blood lipid level (mg/Dl), low-density lipoprotein (LDL) cholesterol, high-density lipoprotein (HDL) cholesterol, and triglyceride values were recorded. The follow-ups of the patients during their hospitalization were examined. Requirement of intensive care hospitalization, central nervous system (CNS) complications (edema or hemorrhagic transformation), the need for mechanical ventilation, the need for decompressive surgery, and mortality were examined and grouped. The discharge status of the patients was analyzed by dividing them into independent (mRS = 0, 1, 2) and dependent groups (mRS = 3, 4, 5).

### Statistical Analysis

The data were analyzed with the SPSS 18.0 Package Software Program (Statistical Package for the Social Sciences Inc.; Armonk, NY, USA). Categorical data were presented as numbers (*n*) and percent (%), and numerical data were presented as mean ± SD (minimum–maximum). The data were assumed to be normally distributed according to the central limit theorem. The chi-square (χ^2^) test was used to compare categorical data. Two independent groups were analyzed with the Independent Sample *t*-test, and more than two groups were analyzed with the one-way ANOVA test. The Bonferroni correction was performed with the Tukey test for *post-hoc* analysis. The relationship between two numerical variables was examined by Pearson's or Spearman's Correlation analysis. Spearman's rho correlation coefficients were accepted as 0.05–0.30, weak; 0.30–0.40, weak-moderate; 0.40–0.60, moderate; 0.60–0.70, strong; 0.70–0.75, very strong; and 0.75–1.00, perfect correlation. The effect of the variables on mortality and disability status was analyzed by the Binary and/or Ordinal Logistic Regression analysis. An ordinal regression analysis was indicated by mRS from 0 up to 5. Patients with mRS = 6 (death) were not included in the ordinal regression analysis. The results were evaluated at the 95% CI, and the statistical significance level was as *p* < 0.05.

## Results

Data from 733 female patients with ischemic stroke with a mean age of 69.53 ± 14.51 years and 858 male patients with stroke with a mean age of 64.27 ± 13.29 years were analyzed for this study. The most common comorbid chronic disease was hypertension (women = 68.5%, men = 53.6%). The most common presenting symptom of the patient was motor deficiency (women = 81.1%, men = 83.2%). The demographic characteristics, chronic diseases, presenting symptoms, ischemia localization, and treatment characteristics of the patients are shown in Table 1.

**Table 1:** Chronic diseases, presenting symptoms, and affected cerebral localizations in all patients with ischemic stroke.

	**Female (*n* = 733)**	**Male (*n* = 858)**	***p*-value**
Age (year), mean ± SD	69.53 ± 14.51	64.27 ± 13.29	0.001*^a^
Scores, mean ± SD			
NIH stroke scale	8.20 ± 7.20	6.30 ± 5.35	0.001*^a^
Glasgow coma Scale	13.67 ± 2.48	14.08 ± 2.02	0.001*^a^
SEDAN score	2.05 ± 1.23	1.56 ± 1.15	0.001*^a^
THRIVE score	3.20 ± 1.87	2.27 ± 1.59	0.001*^a^
Modified Rankin Scale	3.04 ± 1.69	2.55 ± 1.68	0.001*^a^
Chronic diseases, *n* (%)			
Diabetes mellitus	297 (40.5)	244 (28.4)	0.001*^b^
Hypertension	502 (68.5)	460 (53.6)	0.001*^b^
Coronary artery disease	188 (25.2)	258 (30.1)	0.057^b^
Malignancy	46 (6.3)	61 (7.1)	0.547^b^
Chronic renal failure	31 (4.2)	39 (4.5)	0.807^b^
Dementia	44 (6.0)	15 (1.7)	0.001*^b^
Hyperlipidemia	64 (10.5)	45 (6.6)	0.048*^b^
Atrial fibrillation	208 (28.4)	119 (13.9)	0.001*^b^
Smoking, *n* (%)			
Yes	7 (0.9)	75 (8.7)	0.001*^b^
No	736 (99.1)	783 (91.3)	
Initial symptoms, *n* (%)			
Consciousness			
Alert	499 (68.1)	659 (76.8)	0.001*^b^
Other (confused, somnolence, stupor, or coma)	234 (31.9)	199 (23.2)	
Speech			
Normal	370 (43.1)	268 (36.6)	0.216^b^
Aphasia or dysarthria	363 (56.9)	590 (63.4)	
Cranial nerve involvement			
Yes	245 (33.4)	276 (32.2)	0.630^b^
No	488 (66.6)	582 (67.8)	
Motor deficiency			
Yes	587 (81.1)	714 (83.2)	0.512^b^
No	146 (19.9)	144 (16.8)	
Site of vessel occlusion			
Right	438 (59.8)	502 (58.6)	0.356^b^
Left	295 (40.2)	356 (41.4)	
Treatment			
IV thrombolysis	47 (6.4)	77 (9.0)	0.229^b^
Endovascular thrombectomy	36 (4.9)	49 (5.7)	0.224^b^
IV thrombolysis and thrombectomy	44 (6.0)	49 (5.7)	0.504^b^

*Statistically significant value. SD, Standard deviation; IV, Intravenous.

*^a^Independent Sample T-test*.

*^b^Chi-square (χ^2^) test*.

The need for intensive and mechanical ventilation and the length of hospitalization was higher in female patients (*p* = 0.003; *p* = 0.002; *p* = 0.003, respectively). At the same time, mortality and rates of dependency at discharge were higher in female patients (*p* = 0.001). The frequency of CNS complications and the need for decompressive surgery were similar according to gender (*p* = 0.801; *p* = 0.519, respectively). The prognostic characteristics of the patients according to the gender variable are shown in Table 2.

**Table 2:** Evaluation of prognostic status according to gender.

	**Female (*n* = 733)**	**Male (*n* = 858)**	***p*-value**
Length of hospitalization			
Mean ± SD (Minimum–maximum)	12.63 ± 10.95 (1–87)	11.81 ± 12.55 (1–155)	0.003*^a^
Need for intensive care			
Yes	342 (46.7%)	336 (39.2%)	0.003*^b^
No	391 (53.3%)	522 (60.8%)	
Need for mechanical ventilation			
Yes	147 (20.1%)	123 (14.3%)	0.002*^b^
No	586 (79.9%)	735 (85.7%)	
Complication of central system			
Yes	71 (%9.7%)	87 (10.1%)	0.801^b^
No	662 (90.3%)	771 (89.9%)	
Decompressive surgery			
Yes	26 (3.5%)	37 (4.3%)	0.519^b^
No	707 (96.5%)	821 (95.7%)	
Exitus			
Yes	134 (18.3%)	103 (12.0%)	0.001*^b^
No	599 (81.7%)	755 (88.0%)	
Discharge disability			
Dependent	243 (40.7%)	199 (26.4%)	0.001*^b^
Independent	354 (59.3%)	556 (73.6%)	

*SD, Standard deviation*.

*Statistically significant value.

*^a^Independent Sample T-test*.

*^b^Chi-square (χ^2^) test*.

*Data are expressed as numbers (percentage) unless otherwise indicated*.

The relationship between the NIHSS score, the GCS score, SEDAN score, THRIVE score, mRS score, and blood parameters and length of stay in the hospital was investigated in female patients with acute ischemic stroke. The mean hospitalization time was 12.63 ± 10.95 (1–87 days). A low to moderate positive correlation was determined between the length of stay and the NIHSS and mRS scores (Spearman's rho = 0.339 and 0.351; *p* = 0.001 and 0.001, respectively). At the same time, a low level of positive correlation was found between hospitalization time and SEDAN score, THRIVE score, and leukocyte level; and a negative correlation was found between the GCS score and the albumin value (all *p* = 0.001; Spearman's rho = 0.143, 0.211, 0.092, −0.260, −0.234, respectively).

Three hundred forty-two female patients (46.7%) had intensive care admissions. The NIHSS score, SEDAN score, THRIVE score, the mRS, glucose, leukocytes, urea, and CRP values were found to be statistically significantly higher in patients hospitalized in the intensive care unit (*p* = 0.001; *p* = 0.001; *p* = 0.001; *p* = 0.001; *p* = 0.048; *p* = 0.001; *p* = 0.001; *p* = 0.001, respectively). GCS and albumin values were lower in these patients (*p* = 0.001). The NIHSS, SEDAN score, THRIVE score, mRS, and leukocyte and CRP values were found to be statistically significantly higher in female patients who developed CNS complications (*n* = 71, 9.7%) (*p* = 0.001; *p* = 0.001; *p* = 0.001; *p* = 0.001; *p* = 0.005; *p* = 0.015, respectively). GCS values were lower in these patients (*p* = 0.001). Female patients who needed ventilation (*n* = 147, 20.1%) had higher NIHSS score, SEDAN score, THRIVE score, mRS score, blood glucose, creatinine, leukocytes, urea, and CRP values (*p* = 0.001; *p* = 0.001; *p* = 0.001; *p* = 0.001; *p* = 0.008; *p* = 0.001; *p* = 0.001; *p* = 0.001; *p* = 0.001, respectively). GCS and albumin values were lower in these patients (*p* = 0.001). Female patients with decompressive surgery (*n* = 26, 3.5%) had higher NIHSS, SEDAN, THRIVE, mRS scores, and leukocyte values than patients without decompressive surgery (*p* = 0.001; *p* = 0.001; *p* = 0.001; *p* = 0.001; *p* = 0.002, respectively). Female patients who underwent decompressive surgery had lower GCS values (*p* = 0.001).

Mortality rate in female patients was 18.3% (*n* = 134). The GCS score was lower in female patients with fatal outcomes while all other scores were higher (*p* = 0.001). In this patient group, blood glucose, creatinine, leukocytes, urea, and CRP were higher; albumin and hemoglobin values were lower (*p* = 0.009; *p* = 0.001; *p* = 0.001; *p* = 0.001; *p* = 0.001; *p* = 0.001; *p* = 0.001, respectively). Mortality rate, stroke scores, laboratory parameters, and treatment characteristics of female patients according to their mortality status are shown in Table 3.

**Table 3:** Stroke scores and laboratory parameters according to mortality status in female patients with ischemic stroke.

	**Mortality (*n* = 134)**	**Non-mortality (*n* = 599)**	***p*-value**
NIHSS	17.21 ± 7.09 (1–35)	6.19 ± 5.48 (0–25)	0.001*^a^
GCS	10.29 ± 3.45 (3–15)	14.42 ± 1.33 (7–15)	0.001*^a^
SEDAN score	2.91 ± 1.16 (0–6)	1.84 ± 1.15 (0–6)	0.001*^a^
THRIVE score	5.07 ± 1.64 (0–9)	2.79 ± 1.65 (0–8)	0.001*^a^
mRS	4.60 ± 0.83 (1–5)	2.69 ± 1.63 (0–5)	0.001*^a^
Blood glucose (mg/dL)	169.71 ± 88.54 (75–718)	152.61 ± 74.67 (71–558)	0.009*^a^
Creatinine (mg/dL)	1.06 ± 0.56 (0.39–3.69)	0.88 ± 0.57 (0.34–6.26)	0.001*^a^
Hemoglobin (g/dL)	12.26 ± 1.68 (8.20–16.90)	12.65 ± 1.72 (4.6–21.9)	0.020*^a^
Leukocyte (K/uL)	11.14 ± 5.23 (2.50–40.10)	9.00 ± 5.08 (1.4–107.8)	0.001*^a^
Urea (K/uL)	58.94 ± 28.04 (16–165)	44.11 ± 23.34 (11–228)	0.001*^a^
Albumin (g/dL)	2.94 ± 0.61 (1.5–4.30)	3.40 ± 0.50 (1.8–4.4)	0.001*^a^
C-reactive protein (mg/dL)	92.58 ± 98.71 (2.2–470.0)	41.38 ± 66.58 (0.3–476)	0.001*^a^
LDL-cholesterol (mg/dL)	114.80 ± 33.13 (59–191)	126.25 ± 41.09 (37–301)	0.150^a^
HDL-cholesterol (mg/dL)	44.92 ± 16.59 (20–91)	42.68 ± 10.58 (17–96)	0.643^a^
Triglyceride (mg/dL)	143.50 ± 58.01 (49–330)	160.59 ± 93.67 (40–627)	0.052^a^
Treatment (*n*, %)			
IV thrombolysis	15 (11.2)	32 (5.3)	0.841^b^
Endovascular thrombectomy	11 (8.2)	25 (4.2)	
IV thrombolysis and thrombectomy	16 (11.9)	28 (4.7)	

*NIHSS, NIH stroke score; GCS, Glasgow coma score; mRS, Modified Rankin score; LDL, Low-density lipoprotein; HDL, High-density lipoprotein; n, Number; IV, Intravenous.*

*Statistical significance value.

*^a^Independent Sample T-test*.

*^b^Chi-square (χ^2^) test*.

*Data are shown as mean ± SD (minimum–maximum) unless otherwise indicated*.

Female patients who were dependent at discharge (*n* = 243, 40.7%) had lower GCS and albumin values but higher values for all the remaining scores and parameters (urea, leukocytes, CRP) (*p* = 0.001). The stroke scores, laboratory parameters, and treatment characteristics of the female patients according to their depending status are shown in Table 4.

**Table 4:** Stroke scores and laboratory findings according to short-term disability status in female patients with ischemic stroke.

	**Independent (*n* = 354) (mRS = 0–2)**	**Dependent (*n* = 243) (mRS = 3–5)**	***p*-value**
NIHSS	3.01 ± 2.96 (0–22)	10.77 ± 5.05 (2–25)	0.001*^a^
GCS	14.90 ± 0.41 (8–15)	11.72 ± 1.82 (5–15)	0.001*^a^
SEDAN score	1.50 ± 1.10 (0–6)	2.26 ± 1.08 (0–5)	0.001*^a^
THRIVE score	2.22 ± 1.32 (0–5)	3.60 ± 1.75 (0–8)	0.001*
mRS	1.62± 1.15 (0–5)	4.24 ± 0.74 (3–5)	0.001*^a^
Blood Glucose (mg/dL)	149.86 ± 76.26 (73–558)	156.85 ± 74.04 (71–534)	0.016*
Creatinine (mg/dL)	0.89 ± 0.60 (0.37–6.26)	0.88 ± 0.56 (0.34–5.55)	0.828^a^
Hemoglobin (g/dL)	12.66 ± 1.61 (4.6–16.3)	12.66 ± 1.88 (7.50–21.9)	0.793^a^
Leukocyte (K/uL)	8.71 ± 5.95 (1.4–107.8)	9.44 ± 3.43 (3.4–26.8)	0.001*^a^
Urea (K/uL)	41.84 ± 21.42 (13–221)	47.46 ± 25.64 (11–228)	0.001*^a^
Albumin (g/dL)	3.57 ± 0.39 (2.3–4.3)	3.30 ± 0.53 (1.8–4.4)	0.001*^a^
C-reactive protein (mg/dL)	31.61 ± 58.72 (0.3–476)	48.99 ± 71.40 (1.11–471)	0.002*^a^
LDL-cholesterol (mg/dL)	124.75 ± 42.90 (49–301)	129.30 ± 37.64 (37–249)	0.080
HDL-cholesterol (mg/dL)	43.17 ± 10.11 (24–96)	41.94 ± 11.33 (17–73)	0.196^a^
Triglyceride (mg/dL)	167.35 ± 98.08 (48–627)	158.14 ± 84.42 (49–459)	0.082^a^
Treatment (*n*, %)
IV thrombolysis	16 (4.5)	15 (6.2)	0.016*^b^
Endovascular thrombectomy	4 (1.1)	21 (8.6)	
IV thrombolysis and thrombectomy	8 (2.3)	20 (8.2)	

*NIHSS, NIH stroke score; GCS, Glasgow coma score; mRS, Modified Rankin score; LDL, Low-density lipoprotein; HDL, High-density lipoprotein; n, Number; IV, Intravenous.*

*Statistical significance value.

*^a^Independent Sample T test*.

*^b^Chi-square (χ^2^) test*.

*Data are shown as mean ± SD (minimum–maximum unless otherwise indicated)*.

The relationship between treatment and short-term prognosis was evaluated in female patients with ischemic stroke. Mortality was similar in the treatment groups (intravenous thrombolysis = group 1; endovascular thrombectomy = group 2; intravenous thrombolysis + endovascular thrombectomy = group 3) (*p* = 0.841). However, the frequency of independency at discharge was higher in all treatment groups (intravenous thrombolysis; endovascular thrombectomy; intravenous thrombolysis + endovascular thrombectomy) (*p* = 0.016). Logistic regression analysis was performed according to the model established by the NIHSS, GCS, SEDAN scores, THRIVE score, and the mRS score of female patients to predict the mortality. Model fit was good (Nagelkerke R Square = 0.49). According to the results of this analysis, one unit increase in the GCS score reduced the risk of mortality 0.61 times (*p* = 0.001, adjusted OR = 0.611, %95 CI = 0.534–0.701). An increase of one unit in the SEDAN score increases the mortality risk by 1.30 times (*p* = 0.031, adjusted OR = 1.307, 95%CI = 1.024–1.669). An increase of one unit in the mRS score increases the mortality risk by 1.42 times (*p* = 0.011, adjusted OR = 1.424, 95%CI = 1.085–1.870).

The logistic regression analysis was performed in the model established by female patients' NIHSS, GCS, SEDAN, and THRIVE scores to predict the dependency status [mRS 0–2 = functional independence, mRS 3–5 = functional dependency]. Model fit was good (Nagelkerke R Square = 0.610). According to the results of this analysis, one unit increase in the NIHSS increased the risk of being fully dependent by 1.61 times (*p* = 0.001, adjusted OR = 1.611, 95% CI = 1.474–1.762). An ordinal regression analysis was also performed in the model established by female patients' NIHSS, GCS, SEDAN, and THRIVE scores to predict the dependency status. Model fit was good (Nagelkerke R Square = 0.735). According to the results of this analysis, one unit increase in the NIHSS increased the risk of being fully dependent by 1.72 times (*p* = 0.001, adjusted OR = 1.726, 95% CI = 1.612–1.847). The prediction of the mortality status and dependency at the discharge with initial disability scores are shown in Table 5.

**Table 5:** Prediction of the mortality status and dependency at the discharge with initial disability scores.

**Parameter**	**Exp (B)**	**Standard error (SE)**	**95% Confident interval**		***p***
			**Lower**	**Upper**	
Prediction for mortality					
GCS*^a^	0.611	0.070	0.534	0.701	0.001
SEDAN*^a^	1.307	0.125	1.024	1.669	0.031
mRS*^a^	1.424	0.139	1.085	1.870	0.011
Prediction for dependency					
NIHSS**^a^	1.611	0.045	1.474	1.762	0.001
NIHSS***^b^	1.726	0.035	1.612	1.847	0.001

*GCS, Glasgow coma score; mRS, Modified Rankin score; NIHSS, NIH stroke score*.

^*^Model established with by the NIHSS, the GCS, the SEDAN, THRIVE, and the mRS score to predict mortality.

**Model established with the NIHSS, the GCS, SEDAN, and THRIVE scores to predict dependency (independent or dependent).

***Model established with the NIHSS, the GCS, SEDAN, and THRIVE scores to predict dependency (mRS: 0–5).

*^a^Logistic regression analysis*.

*^b^Ordinal regression analysis*.

## Discussion

Although the etiology, mechanisms, and risk factors of diseases have been mostly understood, estimating the prognosis of diseases is one of the most important issues in medicine due to the existence of several relevant factors such as common comorbidities, habits, genetic, and environmental factors. It is important to predict the prognosis in patients with stroke because of high mortality and morbidity. Studies are generally conducted on all patients with stroke. However, the prognostic factors for the female gender, which has specific risk factors, should also be investigated. Women have a stroke at a later age than men and had comorbid diseases during stroke more commonly than men (8); it is known that these factors have an impact on prognosis. Especially in western countries, stroke develops at a younger age; while in countries such as China and Iran, stroke occurs at an older age (16–18). The mean age of female patients with stroke in our study was 68.97 ± 14.61 years. This situation showed us that stroke in women in Turkey occurs in middle-advanced ages. At the same time, female patients with ischemic stroke were older than male patients. When the literature is evaluated in terms of comorbid diseases, the most common chronic disease in women and men is hypertension. Diabetes mellitus and atrial fibrillation are the other most frequent accompanying risk factors (8, 19). In our study, similar to the literature, the most common comorbid chronic diseases in female patients with stroke were hypertension (68.9%) and diabetes mellitus (41.4%). Stroke is a disease with high mortality and disability. The mortality rate in patients varies between 8 and 20%. This rate is quite high, and deaths occur frequently in the first year (7, 19). In our study, the in-hospital mortality rate was determined as 18.3% in women and 12.0% in men, and it was relatively higher in female patients. Considering that stroke-related mortality is higher in female patients in the literature, findings consistent with the literature were also determined in our study (5). The worse prognosis in female patients with stroke suggested that there is a relationship between gender and the severity of stroke.

Many clinical scales are frequently used in the selection of treatment, clinical follow-up, and prognosis in stroke. The most frequently used ones among these are the NIHSS and mRS scores. Another frequently used scale is the GCS, which is used especially in intensive care units. SEDAN and THRIVE scores are the other scales that have been determined to have prognostic importance in the current literature. Decreased GCS and all other increased scores are associated with stroke severity (11–15). In the literature, studies using scales for prognostic evaluation generally included all patients with stroke. Studies in which gender-specific evaluation is performed are rare (20, 21). In our study, NIHSS, SEDAN, and THRIVE scores were higher in female patients who had a fatal outcome, were discharged as functionally dependent, required intensive care unit admission, developed CNS complications, and needed ventilation, while the GCS score was lower. Since scores of these scales can predict prognosis and the need for intensive care in female stroke patients, these scales should also be evaluated for treatment options.

Prolonged hospital stay is a poor prognostic indicator in patients with stroke. Patients with severe stroke tend to stay in the hospital for a long time. This situation increases rates and negatively affects the prognosis of stroke. Therefore, individuating outcome predictors in these patients can guide the disease and treatment process (22, 23). In our study, a statistically significant correlation was found between the length of hospital stay and all scales, while the highest correlation coefficients were with the NIHSS and the mRS. Higher scores on these scales were associated with longer hospital stays.

Changes in hematological values can predict stroke prognosis and may be associated with mortality and disability (24, 25). In particular, leukocyte count changes are frequently affected by many clinical conditions, especially infection and inflammation. However, studies on patients with stroke have shown that the leukocyte count can be an important blood marker in predicting mortality and prognosis (24, 26). Elevated serum glucose level has been determined as a strong indicator of poor prognosis in stroke (27). While glucose elevation is an important risk factor in etiology, especially in patients with diabetes mellitus, it is also associated with increased cerebral ischemic volume and poor functional status (28). As a result, it was determined that hyperglycemia was associated with the size of the lesion and the severity of the neurological deficit (29). It has been shown that there is a relationship between high renal function tests and low serum albumin levels and prognosis, and increased values are associated with poor functional outcomes (30). While high serum lipids can be an important risk factor in the etiology of stroke, it has been stated that high serum cholesterol levels can be associated with favorable outcomes (31). In this study, it was shown that there is a relationship between high inflammatory values (especially leukocytes and C-reactive protein) and poor in-hospital prognosis. The elevated blood glucose level at admission was associated with increased mortality and the need for ventilation. Similarly, increased serum urea and creatinine levels were associated with mortality. Low albumin level was also found to be an indicator of poor in-hospital prognosis. Urea and albumin levels were associated with disability at discharge; indeed, the increased value of urea and the decreased value of albumin were associated with worse functional outcomes. There was no relationship between blood lipid levels and prognosis. These prognostic markers should be confirmed with multicenter studies.

In conclusion, female patients with ischemic stroke of anterior circulation have a worse short-term prognosis, higher mortality, and a higher rate of disability. The NIHSS and the mRS can be used to predict prognosis, higher mortality, and disability. Although the NIHSS and the mRS can be used to predict prognosis in these patients, the GCS, SEDAN, and THRIVE scores are also important measures in short-term prognosis. In addition, higher levels of blood glucose, creatinine, urea, leukocytes, and CRP and lower levels of albumin and hemoglobin predict poor prognosis.

This study has several limitations. First, this is a retrospective and cross-sectional study. Second, the national and geographic features could not be evaluated because data from a single center were used. Third, the patients were not divided into groups according to ischemic stroke localization (frontal, temporal, etc.). Fourth, the serum markers evaluated in the study are frequently affected by many clinical and structural conditions. Fifth, female patients were not divided into groups according to their menstrual cycle, pregnancy, or menopause status. Sixth, long-term (3rd month, 6th month, or 1st year) prognosis and causes of mortality were not assessed. Seventh, factors, such as infection, that might have an adverse impact on morbidity and mortality during hospitalization (pneumonia, etc.) were not evaluated. Importantly, other variables, such as stroke subtype, the presence of vessel occlusion, lesion volume, the collateral circulation status, and recanalization, were not taken into account. Multivariate analyses for mortality and dependency were adjusted only for the stroke scales, which were under investigation in this study for prognosis prediction (NIHSS, mRS, GCS, SEDAN, and THRIVE). Additionally, blood parameters and disability scores of male patients with ischemic stroke were not evaluated for comparison.

## Data Availability Statement

The raw data supporting the conclusions of this article will be made available by the authors, without undue reservation.

## Ethics Statement

The studies involving human participants were reviewed and approved by Selcuk University Clinical Research Local Ethics Committee (approval number: 2020-473).

## Author Contributions

FE: planning, organization, writing, and editing. SO: planning, organization, and editing. CO: planning, organization, data collection, writing, and editing. All authors contributed to the article and approved the submitted version.

## Conflict of Interest

The authors declare that the research was conducted in the absence of any commercial or financial relationships that could be construed as a potential conflict of interest.

## Publisher's Note

All claims expressed in this article are solely those of the authors and do not necessarily represent those of their affiliated organizations, or those of the publisher, the editors and the reviewers. Any product that may be evaluated in this article, or claim that may be made by its manufacturer, is not guaranteed or endorsed by the publisher.
